# A Comprehensive Analysis of Y-Chromosome Microdeletions and Their Relationship to Male Infertility and Lifestyle Variables

**DOI:** 10.7759/cureus.57375

**Published:** 2024-04-01

**Authors:** Manisha B Sinha, Dharam S Rathia, Rima Dada, Human P Sinha

**Affiliations:** 1 Anatomy, Laboratory of Cytogenetics and Molecular Reproduction, All India Institute of Medical Sciences Raipur, Raipur, IND; 2 Anatomy, All India Institute of Medical Sciences, Raipur, Raipur, IND; 3 Anatomy, Laboratory for Molecular Reproduction and Genetics, All India Institute of Medical Sciences New Delhi, New Delhi, IND; 4 Neurology, Narayana Health-Memon Medical Institute (NH-MMI) Superspeciality Hospital, Raipur, IND

**Keywords:** life style, age and ageing, male factor infertility, body mass index: bmi, y-chromosome microdeletion

## Abstract

Background: Male infertility is the inability of a male to conceive a fertile female during at least a year of unprotected sexual activity. A variety of medical conditions and treatments cause male infertility. Y chromosome microdeletion is an important cause of infertility among males. Various epidemiological factors also play a role in the occurrence of infertility. Our study aims to determine the association between Y-chromosome microdeletion and age, sperm count, body mass index (BMI), alcohol, and tobacco consumption.

Methods: This study was conducted in 70 male infertility cases. Data was collected from 2018 to 2023 at the Genetic Lab, Department of Anatomy, All India Institute of Medical Sciences, Raipur, Chhattisgarh, India. Demographic profiles, including age, sperm count, weight and height, and history of smoking and drinking, were collected from individuals. BMI was calculated, and chromosome analysis was done for Y chromosome microdeletion. Both multiplex and singleplex methods were used to determine the microdeletion using a thermocycler (Applied Biosystems, Veriti^TM^ 96-well Fast Thermal Cycler, 0.2 ml USA) in AZF, and the association between age, sperm count, BMI, alcohol, and tobacco was determined.

Results: The number of regions deleted among individuals varies from one to seven. Regions Sy746, Sy143, and Sy145 were found to be commonly deleted. We found a positive, but not statistically significant, correlation between age and microdeletion (point biserial correlation coefficient (r) = 0.2, p-value = 0.097). When comparing age with sperm count, the results showed a negative correlation, highlighting the influence of age on sperm count (r (68) = 0.284, p = 0.017). In comparing BMI and microdeletion, no significant relationship (χ² = 3.7, p = 0.296) indicated independence between them. According to our observations, microdeletion affects all smokers and 45% of non-smokers. We found a significant association between smoking and microdeletion (χ^2^ = 4.49, P = 0.034). There was no statistically significant relationship between microdeletion and drinking (χ²(3) = 5.65, p = 0.13).

Conclusion: We discovered a significant positive association between smoking and a positive, but not statistically significant, correlation between age, BMI, and drinking, as well as a microdeletion. There are probably a lot of unidentified variables that affect successful fertilization and implantation. These could include variables that affect fertility and the success of reproduction on an environmental, genetic, and epigenetic level. The study reveals that Y chromosome microdeletion and other epidemiological factors coexist concurrently in cases of infertility. Assessing these variables is crucial for infertile patients. A community-based, comprehensive survey is required to assess the overall consequences of various epidemiological factors on infertility.

## Introduction

Male infertility is the inability of a male to conceive a fertile female during at least a year of unprotected sexual activity [1]. Approximately 20% of infertility cases involve only the male partner [2]. Y chromosome deletions and microdeletions are thought to affect roughly one in every 2000-3000 births, making them a rare cause of infertility [3].

Male infertility can be caused by a variety of medical conditions and treatments, including hormonal imbalances, varicoceles, blockage of the reproductive tract leading to abnormalities in the ejection of semen, and chemotherapy [2,4]. Lifestyle choices like heavy alcohol consumption and smoking can impact fertility [5-7].

Worldwide, researchers have attempted to identify the relationship between various factors like age, alcohol and tobacco use, BMI, and hormonal abnormalities, as well as how it affects sperm quantity and quality in cases of male infertility [5,6,8-12]. Only a few researchers have tried to determine these factors’ impact on Y-chromosome microdeletion, but the precise correlation has not been determined [10,13].

A more profound understanding of the relationship between Y-chromosome microdeletions and deletions and other variables will be extremely helpful in creating and carrying out preventative and treatment plans. Our study aims to determine the association between sperm count, Y-chromosome microdeletion, age, BMI, alcohol, and tobacco.

## Materials and methods

Study population

We examined 70 male known cases of infertility, including azoospermia, oligozoospermia, and severe oligozoospermia. Data was collected from 2018 to 2023 at the Genetic Lab Department of Anatomy, All India Institute of Medical Sciences (AIIMS), Raipur, Chhattisgarh, India. The age group ranges from 22 to 55 years old. The study was approved by the institute’s ethical committee (AIIMSRPR/IEC/2017/057), and informed consent was obtained before the recruitment of participants.

Data collection

Demographic profiles, including age, sperm count, weight, height, and history of smoking and drinking, were collected from individuals. Body mass index (BMI) was calculated as weight in kilograms and divided by the square of height in meters (Kg/m^2^). Based on BMI, participants were categorized as normal, underweight, overweight, and obese per standard classification [14]. Information on the sperm count was collected from the patient. Based on sperm count (millions/ml^2^), participants were categorized as having azoospermia (0), severe oligozoospermia (<1 million/ml^2^), moderate oligozoospermia (1-5 million/ml^2^), mild oligozoospermia (5-20 million/ml^2^), or normal (>20 million/ml^2^). [15]

Blood sample analysis

Participants’ blood samples (2 ml) were collected in an EDTA vial. Genomic DNA was extracted using a Kit Quizen DNA isolation kit (QIAamp DNA Blood Mini Kit). The primers were synthesized by ACTIVE^TM^ Oligos from ILS (Imperial Life Sciences Pvt. Ltd., Gurgaon, Haryana) [16].

Multiplex and singleplex methods were used to determine the microdeletion using a thermocycler (Applied Biosystems, Veriti ^TM^ 96-well FastThermal Cycler, 0.2 ml, USA).

Multiplex was done with a primer of the following sequence-tagged sites (STS), 

Multiplex A: SRY, Sy86, Sy127, Sy254, and

Multiplex B: SRY, Sy89, Sy143, Sy255. 

For a total volume of 25 µl, the multiplex mixture contained distilled water (13.7 µl), 10X buffer (2.5 µl), dNTP (0.4µl), forward and reverse primers (2-3 µl each), 1000IU Taq polymerase (0.2 µl), and extracted DNA (5 µl). Temperature (Tm) was 59°C-72°C in the thermal cycler. 

Singleplex was done with the primer of the following sequence-tagged sites (STS), and

Sy746, Sy182, Sy121, Sy128, Sy130, Sy135, and Sy160. 

The content for singleplex was as follows: in total, 13 µl, water-9.8 µl, 10X Buffer-1.25 µl, dNTPs-0.15 µl, forward and reverse prime 0.3 µl each, 1000IU Taq-0.2 µl, and extracted DNA-1 µl were taken. Tm for single-plex PCR ranged from 57 to 60°C.

**Figure 1:**
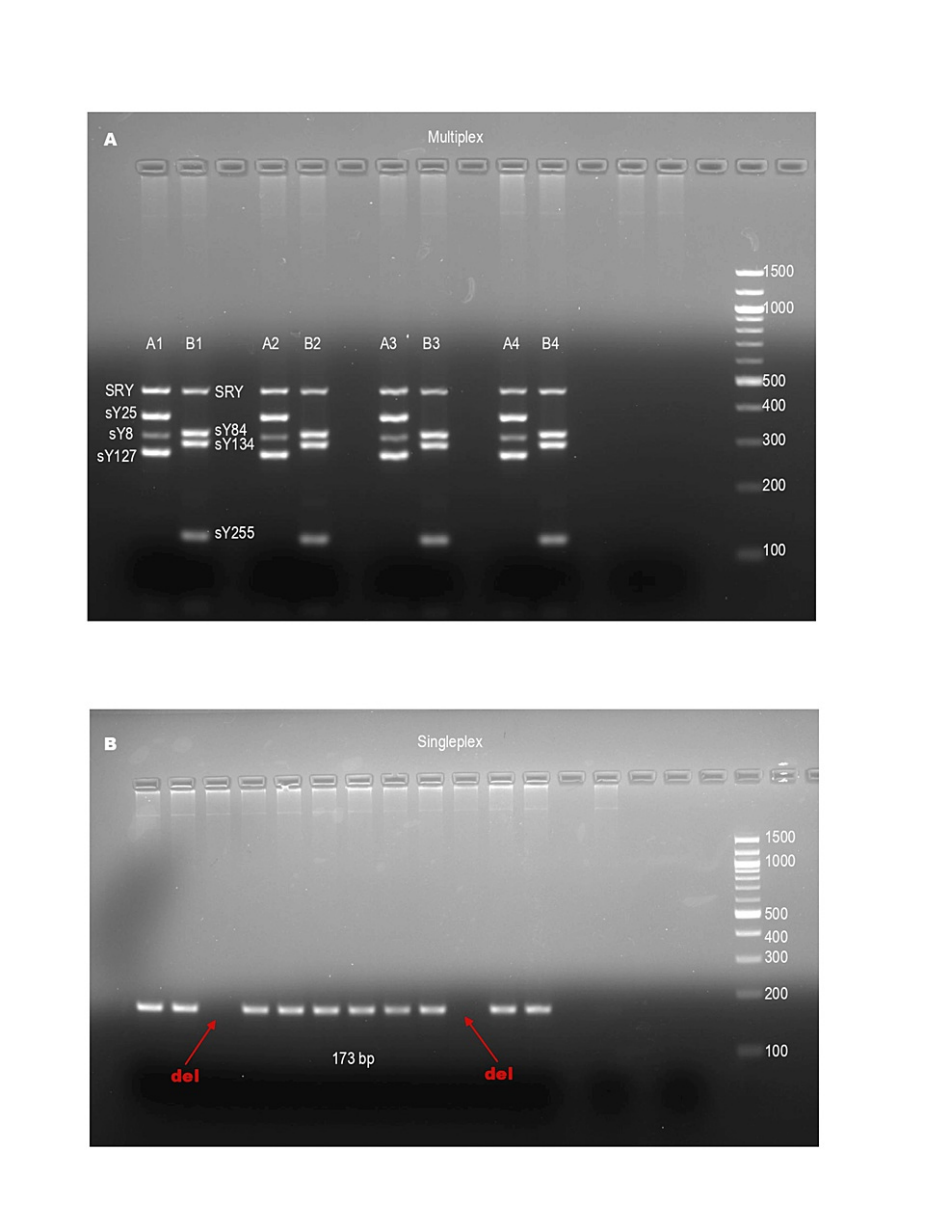
Gel electrophoresis image, A: multiplex of case 1 (A1, B1), case 2 (A2, B2), case 3 (A3, B3), control (positive, A4, B4), control (negative, no band), Ladder, B: Singleplex of Sy 130 of 12 cases showing deletion (del) in two cases

Initial denaturation at 95°C for 5 min was followed by 40 cycles of denaturation at 95°C for 30 min, annealing at 60°C for 5 min, extension at 72°C for 60 s, and a final extension at 72°C for 7 min. After being separated by electrophoresis on a 2% agarose gel, the PCR products were stained with ethidium bromide and exposed to ultraviolet light (Figure 1).

Data pre-processing

Before analyzing the data, we organized it according to inclusion criteria, calculated the BMI from weight and height, and categorized the individuals’ BMI and sperm count using standard classification. Data were analyzed using the statistical software Jamovi (Version 2.3.28.0), Microsoft Excel (Version 16.52), and the online DATA tab [17].

## Results

Descriptive statistics

We collected data, including age, sperm count, weight, height, and history of smoking and drinking, from 70 male individuals. The age group of individuals ranges from 22 to 53 years (mean ± SD: 32.53 ± 6.33). The age of married life ranges from 1 to 20 years (mean ± SD: 5.64 ± 4.39), and the duration of infertility ranges from 0.5 to 13 years (mean ± SD: 2.88 ± 2.64) (Table 1). We measured height and weight and calculated BMI. The calculated BMI ranges from 14.4-34.5 kg/m2 (mean ± SD: 24.44 ± 4.22) (Table 1).

**Table 1:** Descriptive statistics of participants

Descriptive statistics	Age (Years)	BMI (Kg/m^2^)	Age of Married Life (Years)	Duration of Infertility (Years)
Mean	32.53	24.44	5.64	2.88
Std. Deviation	6.33	4.22	4.39	2.64
Minimum	22	14.4	1	0.5
Maximum	53	34.5	20	13
Mean ± Std.	32.53 ± 6.33	24.44 ± 4.22	5.64 ± 4.39	2.88 ± 2.64

**Figure 2:**
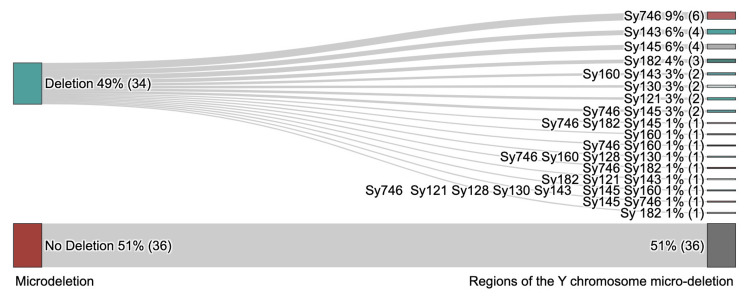
Microdeletion frequency and regions

The number of regions deleted among individuals varies from one to seven. Regions Sy746, Sy143, and Sy145 were common. Analysis revealed micro-deletion multiples of the region of the Y chromosome mentioned in Figure 2.

Inferential statistics

Age vs. Sperm Count

There was a negative correlation between age and sperm count. The Pearson correlation result showed a significant correlation between age and sperm count, r (68) = 0.284, p = 0.017.

Age vs. Microdeletion

There was a positive correlation between age and microdeletion, which was not statistically significant (r = 0.2, n = 70, p =0.097).

Sperm Count and Microdeletion

**Figure 3:**
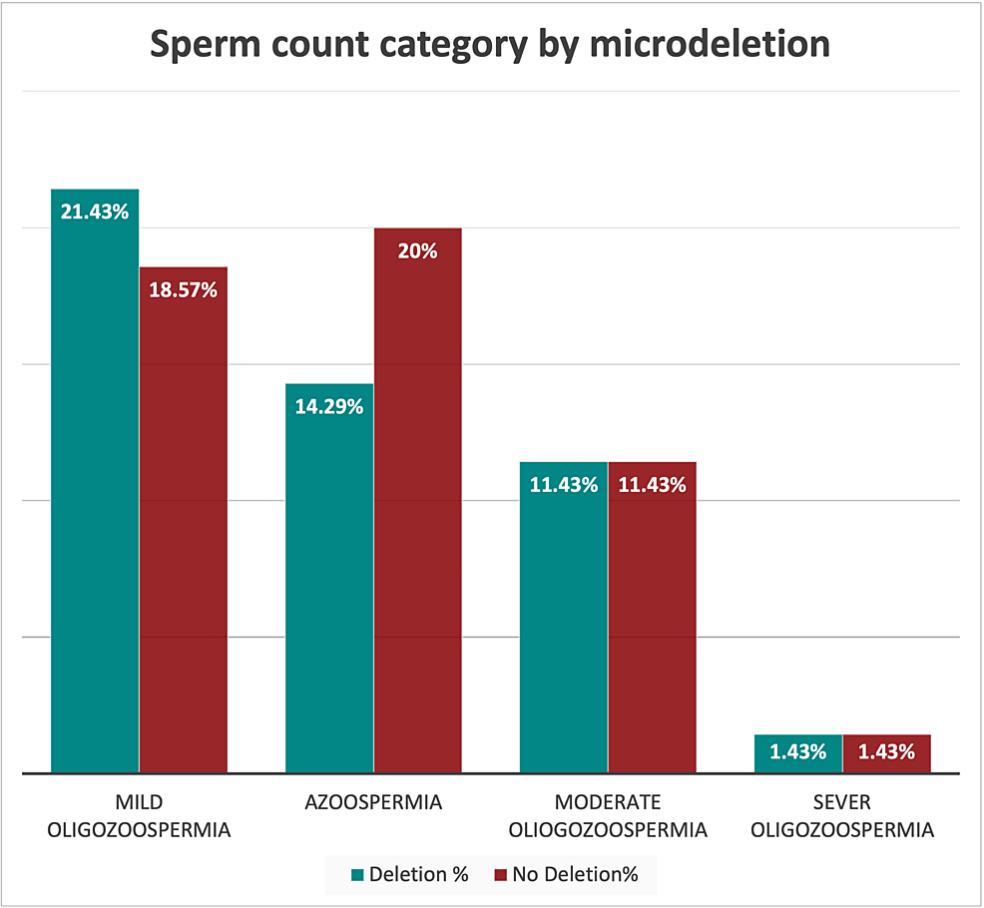
Sperm count category by microdeletion

Of the 70 individuals, 28 (or 40%) had mild oligozoospermia, 24 (or 34.29%) had azoospermia, 16 (or 22.86%) had moderate oligozoospermia, and two (2.86%) had severe oligozoospermia (Figure 3).

The number of individuals having deletions was in the following order: mild oligozoospermia, 15 (21.43%) > azoospermia 10 (14.29%) > moderate oligozoospermia 8 (11.43%) > severe oligozoospermia 1 (1.43%) (Figure 3).

There was no statistically significant relationship between the sperm count category and microdeletion, χ² (3) = 0.75, p = 0.861 (Table 2).

**Table 2:** Chi2 test result and p-value

Association between	Chi^2^	Degree of freedom (Df)	p-value
Sperm count category and microdeletion	0.75	3	0.861
BMI Category and microdeletion	3.7	3	0.296
microdeletion and smoking	4.49	1	0.034
Microdeletion and drinking	5.65	3	0.13

BMI and Microdeletion

**Figure 4:**
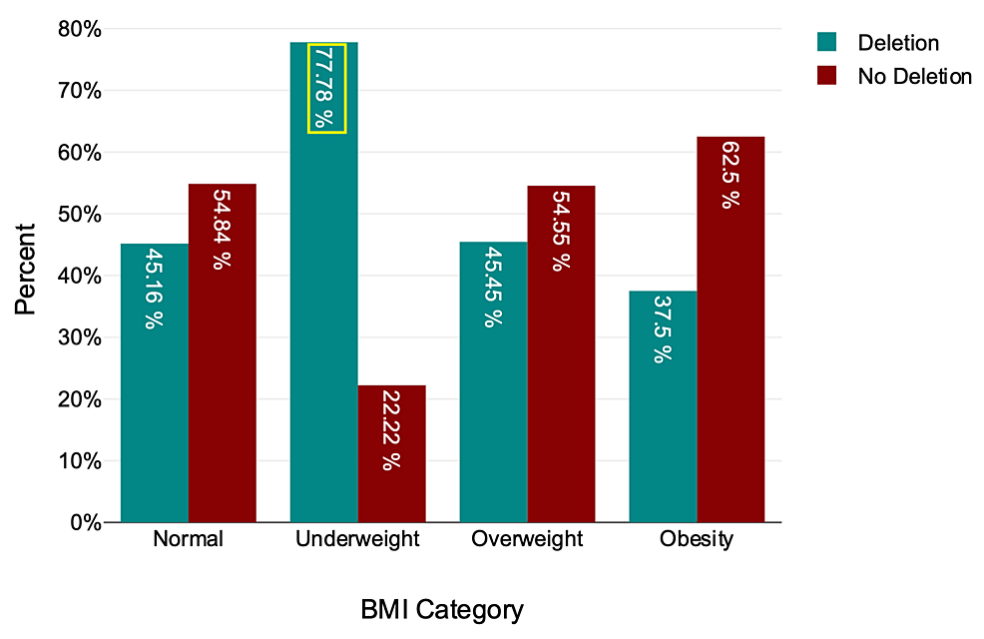
Microdeletion by BMI category has the highest percentage of deletion among the underweight category

Of a total of 70 individuals, 31 (44.29%) had a normal BMI, 22 (31.43%) were overweight, nine (12.86%) were underweight, and eight (11.43%) were obese (Table 3, Figure 4). The frequency of microdeletion within the BMI category is highest among underweight individuals, with seven among nine (77.78%) (Table 3, Figure 4).

**Table 3:** BMI and microdeletion

Frequency of BMI and Microdeletion					
BMI category	Microdeletion				
	Individuals with deletion	Percent (%) within BMI category	Individuals without deletion	Percent (%) within BMI category	Total percent (%)
Normal	14	45.16%	17	54.84%	31 (44.29%)
Underweight	7	77.78%	2	22.22%	9 (31.43%)
Overweight	10	45.45%	12	54.55%	22 (12.86%)
Obesity	3	37.5%	5	62.5%	8 (11.43%)
Total	34		36		70 (100%)

There was no statistically significant relationship between BMI category and microdeletion, χ²(3) = 3.7, P = 0.296 (Table 2). The association between microdeletion and BMI was examined using a point-biserial correlation. A positive correlation between BMI and microdeletion was not statistically significant (r = 0.2, n = 70, p = 0.093).

Smoking and Microdeletion

Out of 70 individuals, 66 (94.29%) were non-smokers, and four (5.71%) were smokers (Table 4).

**Table 4:** Smoking and microdeletion

Smoking and Microdeletion					
Microdeletion	Smoking				
	Smoker	% within smoking	Non-smoker	% within smoking	Total
Deletion	4	100%	30	45.45%	34
No deletion	0	0%	36	54.55%	36
Total	4 (5.71%)	100%	66 (94.29%)	100%	70 (100%)

**Figure 5:**
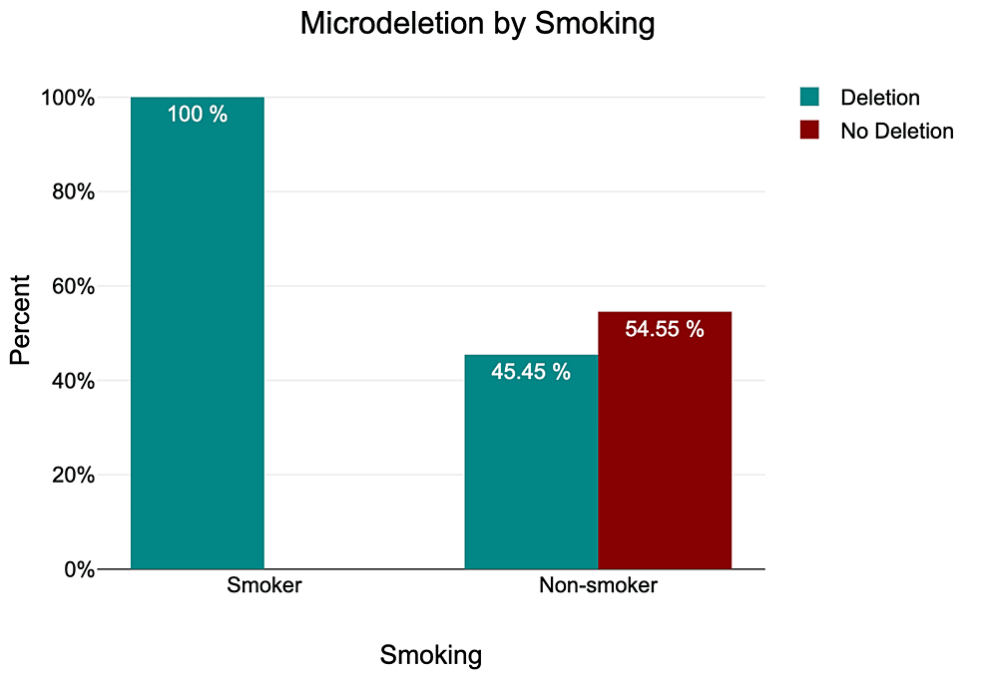
Smoking and microdeletion

According to our observations, microdeletion affects all four smokers and 45% of non-smokers. We found a significant association between smoking and microdeletion (χ²=4.49, P=0.034 ) (Tables 2, 4, and Figure 5).

A Fisher exact test was performed between microdeletion and smoking. There was no statistically significant relationship between microdeletion and smoking (p = 0.05).

Alcohol Consumption and Microdeletion

**Figure 6:**
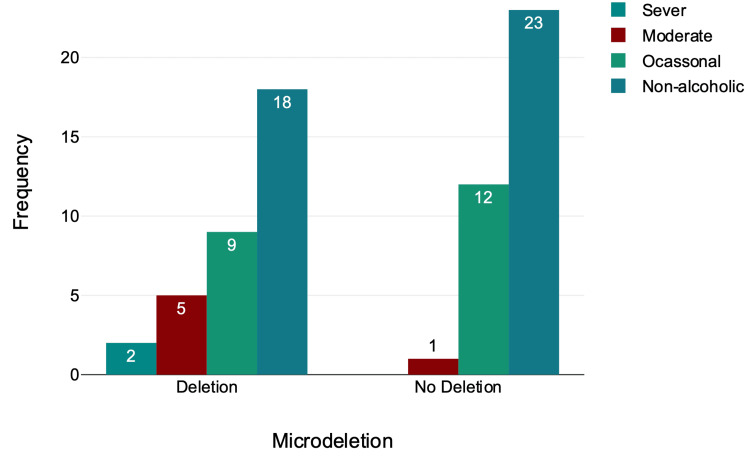
Drinking and microdeletion

Out of 70 individuals, 41 (58.57%) were non-alcoholic, 21 (30%) were occasional drinkers, six (8.57%) were moderate, and two (2.86%) were severe drinkers (Table 5, Figure 6).

Within the group of individuals who drink, 83.33% were moderate drinkers, 42.86% were occasional drinkers with microdeletion, and 43.09% were non-alcoholics with microdeletion in their Y chromosomes (Table 5, Figure 6).﻿

**Table 5:** Drinking and microdeletion

Drinking and Microdeletion					
Alcohol category	Microdeletion				
	Deletion		No Deletion		Total
	n	% within alcohol category	n	% within alcohol category	n
Sever	2	100%	0	0%	2
Moderate	5	83.33%	1	16.67%	6
Occasional	9	42.86%	12	57.14%	21
Non-alcoholic	18	43.9%	23	56.1%	41
Total	34		36		70

There was no statistically significant relationship between microdeletion and drinking, χ²(3) = 5.65, p = 0.13 (Table 5, Figure 6).

## Discussion

We started our study to find an association between microdeletion and age, sperm count, BMI, smoking, and drinking. Only a few researchers have tried to determine the association between these factors and Y-chromosome microdeletion, and the precise correlation has not been determined [10,13,18]. We collected information and samples from 70 male individuals, covering age, weight, height, smoking and drinking history, and infertility-related details. The ages of individuals ranged from 22 to 53 years, and their BMI ranged from 14.4 to 34.5. BMI was calculated, individuals were categorized by BMI sperm count, and their association with microdeletion was compared.

Y-chromosome microdeletion and infertility

Specific genes in the Y chromosome are crucial for developing male reproductive organs [18]. As a result, the Y chromosome plays a direct or indirect role in controlling testosterone production [19]. When genetic material is lost from the Y chromosome long arm, it is called Yq microdeletion [18]. This condition can lead to reduced sperm production and higher chances of infertility [18,19]. Genetic testing can be conducted to identify the presence of Yq microdeletion in a man if needed [18,20]. In this study, the deletion frequency among individuals varies from one to seven regions. Regions Sy746, Sy143, and Sy145 were common.

Age

On comparing age and microdeletion, we found a positive but not statistically significant correlation. The age of the male partner plays an important role in reproduction, as it can lead to higher rates of sperm DNA fragmentation, an increase in acquired medical conditions, and a decline in semen quality and male sexual function [21]. Aging also impacts sperm parameters and fertility, resulting in decreased fecundability, longer conception times, and higher rates of miscarriage [22]. There are still many unknowns about the effects of male aging on fertility [23]. In a study, researchers at a tertiary care center in Eastern India who studied infertile couples between the ages of 21 and 50 did not discover any evidence that the male partner’s semen quality declined with age [24]. Numerous studies have been published, but the relationship between age and semen parameters is still unclear, and the correlation between age and microdeletion has not been studied [21-23]. A more profound comprehension of age and its influence on every aspect of male fertility will be possible with additional research [23].

Sperm count and microdeletion

Comparing age and sperm count, we found a significant positive correlation, emphasizing the impact of age on sperm count. A significant positive correlation between age and sperm count highlights the effect of age on male fertility. However, a study in tertiary care centers in eastern India showed no significant decline in sperm count, and the findings indicated the complex relationship between age and semen quality [22]. On comparing sperm count and microdeletion, no significant relationship indicated independence between sperm count categories. In the current study, the frequency of deletions was higher in mild oligozoospermia 15 (21.43%), azoospermia 10 (14.29%), moderate oligozoospermia eight (11.43%), and severe oligozoospermia one (1.43%) (Figure 3). In another study, a microdeletion score of 16.1% was found in 19 out of 118 cases in a population study in the West, which had AZFc deletions, compared to 33% with AZFa, 7% with AZFb, and 12 per region [24]. In comparison to severe oligozoospermic subjects (2 of 19), male azoospermic subjects (17 of 19) had a higher score [24].﻿

Effect of BMI

BMI measures body fat based on an individual’s height and weight and is commonly used to assess weight status and overall health [14]. Biabangard A. et al. studied 180 males for microdeletions and BMI and found Y-chromosome microdeletion in obese men [10]. Chromosome microdeletion affects testosterone levels, and testosterone levels affect obesity [25,26]. According to research, rising male BMI lowers testosterone and sex hormone-binding globin concentrations, while boosting estrogen levels leads to decreased testosterone and increased estrogen levels [26]. In comparing BMI and microdeletion, no significant relationship indicated independence between microdeletion and BMI. However, a positive correlation with BMI suggests potential trends that warrant further investigation. A study found Yq microdeletion in 8.6% of overweight and 5.3% of obese people [10]. However, in this study, microdeletion is higher among underweights (77.78%).

Effect of smoking and alcohol

Higher sperm motility decreased semen volume, and total sperm count was linked to cigarette smoking; however, semen quality may improve as a result of quitting smoking [27]. Men who smoked were found to have lower sperm counts in a meta-analysis of 20 observational studies [28]. Although smoking tended to decrease semen volume, cigarette smoking had no discernible impact on sperm concentration, motility, or reproductive hormone levels [5]. Other potentially modifiable risk factors, such as BMI, cigarette smoking, using cannabis or street drugs, or smoking cigarettes, were not linked to elevated risk [9]. At higher concentrations, nicotine concentration (100ng/ml) impacts the percentage of viable spermatozoa, accelerates sperm DNA fragmentation, and accelerates spermatozoa apoptosis [29]. Research revealed that an abnormally high intake of alcohol over 60 g per day is highly linked to azoospermia, a condition that may be curable following alcohol withdrawal [11].

We found a significant association between smoking and microdeletion (p=0.034) (Table 2). Alcohol consumption did not show a statistically significant association with microdeletion. A similar trend was seen in a study by Olsen J et al., who mentioned that alcohol consumption shows little effect on fecundability [6]. Other factors need to be considered, as research on the relationship between cigarette smoking and the eight sperm quality parameters evaluated by laser-Doppler spectroscopy and DNA flow cytometry revealed no conclusive evidence of a connection [29].

Implication

Even though we did not find a significant association between lifestyle variables and Y chromosome microdeletion, these factors may be affecting male reproductive health through some other mechanisms. Therefore, more in-depth studies on a larger population will be required to identify. To increase the success rate of infertility treatment, clinicians should adopt a comprehensive strategy and advise patients on lifestyle modifications. Our study has emphasized the necessity of treating male infertility holistically. These results can be used in public health campaigns to raise awareness of lifestyle factors that affect male fertility.

Limitation 

The sample size of 70 participants was small, which may have limited the generalizability of the findings to many populations. Establishing a relationship between variables is challenging because of the cross-sectional design. A more thorough understanding of causal relationships and longitudinal research may be helpful sometimes. Self-reported data, which is subject to recollection and social desirability bias, is the basis for the study’s smoking and drinking history. Despite performing a Y chromosome microdeletion analysis, the investigation did not examine other genetic factors that could be involved in male infertility. The investigation of external factors, such as exposure to the environment and occupational risks, that may impact fertility was not done in depth in this study.

Future work

To put the study’s findings into context, direct future research efforts to overcome these obstacles, and deepen our understanding of male infertility, it is imperative that these limitations be acknowledged. The results indicate potential directions for future investigation to decipher the underlying mechanisms of the observed correlations. Further research could examine the complex relationship among age, lifestyle, and genetic factors in greater detail, laying the groundwork for focused interventions. There is much more work to be done to grasp the complete picture of male infertility.

## Conclusions

The study offers a thorough examination of the variables affecting male infertility. Our result shows that age significantly influences sperm count but is not significantly associated with microdeletion. A positive correlation between BMI and microdeletion was not statistically significant. It appears that smoking is an important factor linked to microdeletion, and there was no discernible correlation between alcohol use and infertility. The complexity of the condition calls for more studies to gain a better understanding. There are probably a lot of unidentified variables that affect successful fertilization and implantation. These could include variables that affect fertility and the success of reproduction on an environmental, genetic, and epigenetic level. The study reveals that Y chromosome microdeletion and other epidemiological factors coexist concurrently in cases of infertility. Assessing these variables is crucial for infertile patients. A community-based, comprehensive survey is required to provide essential details about these connections.
